# A Randomized Phase 4 Study of Immunogenicity and Safety After Monovalent Oral Type 2 Sabin Poliovirus Vaccine Challenge in Children Vaccinated with Inactivated Poliovirus Vaccine in Lithuania

**DOI:** 10.1093/infdis/jiaa390

**Published:** 2020-07-04

**Authors:** Ananda S Bandyopadhyay, Chris Gast, Elizabeth B Brickley, Ricardo Rüttimann, Ralf Clemens, M Steven Oberste, William C Weldon, Margaret E Ackerman, Ruth I Connor, Wendy F Wieland-Alter, Peter Wright, Vytautas Usonis

**Affiliations:** 1 Bill & Melinda Gates Foundation, Seattle, Washington, USA; 2 Biostatistical Consulting, Washington, USA; 3 Department of Infectious Disease Epidemiology, London School of Hygiene & Tropical Medicine, London, United Kingdom; 4 Fighting Infectious Diseases in Emerging Countries, Miami, Florida, USA; 5 Global Research in Infectious Diseases, Rio de Janeiro, Brazil; 6 Division of Viral Diseases, Centers for Disease Control and Prevention, Atlanta, Georgia, USA; 7 Thayer School of Engineering, Dartmouth College, Hanover, New Hampshire, USA; 8 Department of Pediatrics, Geisel School of Medicine at Dartmouth, Dartmouth-Hitchcock Medical Center, Lebanon, New Hampshire, USA; 9 Clinic of Children’s Diseases, Institute of Clinical Medicine, Faculty of Medicine, Vilnius University, Lithuania

**Keywords:** poliovirus, vaccine, inactivated poliovirus vaccine, oral poliovirus vaccine, viral shedding, immunogenicity

## Abstract

**Background:**

Understanding immunogenicity and safety of monovalent type 2 oral poliovirus vaccine (mOPV2) in inactivated poliovirus vaccine (IPV)–immunized children is of major importance in informing global policy to control circulating vaccine-derived poliovirus outbreaks.

**Methods:**

In this open-label, phase 4 study (NCT02582255) in 100 IPV-vaccinated Lithuanian 1–5-year-olds, we measured humoral and intestinal type 2 polio neutralizing antibodies before and 28 days after 1 or 2 mOPV2 doses given 28 days apart and measured stool viral shedding after each dose. Parents recorded solicited adverse events (AEs) for 7 days after each dose and unsolicited AEs for 6 weeks after vaccination.

**Results:**

After 1 mOPV2 challenge, the type 2 seroprotection rate increased from 98% to 100%. Approximately 28 days after mOPV2 challenge 34 of 68 children (50%; 95% confidence interval, 38%–62%) were shedding virus; 9 of 37 (24%; 12%–41%) were shedding 28 days after a second challenge. Before challenge, type 2 intestinal immunity was undetectable in IPV-primed children, but 28 of 87 (32%) had intestinal neutralizing titers ≥32 after 1 mOPV2 dose. No vaccine-related serious or severe AEs were reported.

**Conclusions:**

High viral excretion after mOPV2 among exclusively IPV-vaccinated children was substantially lower after a subsequent dose, indicating induction of intestinal immunity against type 2 poliovirus.

**(See the Major Articles by Yan et al on pages 113–8 and Editorial Commentary by Cochi and Pallansch on pages 7–9.)**

Poliomyelitis is on the verge of global eradication, with only Pakistan and Afghanistan currently reporting wild poliovirus type 1 cases [1]. Eradication of wild-type 2 poliovirus was declared on 20 September 2015 [2], and eradication of wild-type 3 poliovirus on 24 October 2019 [3]. However, in rare cases oral poliovirus vaccine (OPV) viruses can revert to neurovirulence and cause vaccine-associated paralytic poliomyelitis in vaccinees or susceptible contacts. In settings with low immunization coverage, shed vaccine virus can acquire transmissibility and neurovirulence to generate vaccine-derived polioviruses (VDPVs). The OPV type 2 component has been associated with >90% of all circulating VDPVs (cVDPVs) globally [4]. To mitigate against cVDPV and vaccine-associated paralytic poliomyelitis, type 2–containing OPV was withdrawn from routine use and replaced with bivalent OPV containing only types 1 and 3 in May 2016 [5]. This unprecedented globally synchronized switch is the first step toward a sequential withdrawal of all live poliovirus vaccines and replacement by inactivated poliovirus vaccine (IPV) as vaccine of choice for elective polio protection after certification of eradication of wild polioviruses [6].

OPV replicates in the gut and induces primary intestinal immunity, an essential factor to control person-to-person transmission in settings of poor hygiene [7]. IPV induces humoral immunity that provides protection against paralytic poliomyelitis, but limited primary intestinal immunity compared with OPV [8]. Therefore, global IPV-only primary immunization regimens in the postcertification period will eliminate the risk of generating VDPV but will not affect person-to-person viral transmission in settings with cVDPV. This situation is illustrated by the ongoing, expanding type 2 cVDPV outbreaks in multiple countries in Africa and elsewhere for over 4 years after type 2 OPV (OPV2) cessation [4]. For this reason, stockpiles of monovalent OPV (mOPV) must be maintained for outbreak control, for which novel, more genetically stable OPV vaccines are being developed [9]. We conducted the current study knowing that the data generated would be valuable for interpreting other studies with these new vaccines, as understanding the dynamics of mOPV challenge in young children with no prior OPV exposure will be critical for designing the outbreak response strategies and preparing contingency options for the global program.

Before OPV2 cessation, the Lithuanian primary immunization schedule, 3 IPV doses administered in the first year of life, provided a unique opportunity to assess primary immunogenicity from an IPV-only regimen in conditions that mimic the posteradication era. We assessed humoral and intestinal immunity before and after 1 or 2 challenge doses of type 2 mOPV (mOPV2) in 1–5-year-old Lithuanian children, along with assessment of postvaccination viral shedding dynamics.

## METHODS

This open-label, phase 4 study was conducted between 7 January 2016 and 12 May 2016 in 3 outpatient clinics located in Lithuania: Centro Poliklinika and Naujininku Poliklinika, both in Vilnius, and UAB Inmedica in Kaunas. The protocol was approved by the local institutional review board and registered on ClinicalTrials.gov (NCT02582255). The study was performed according to current International Council for Harmonisation and Good Clinical Practice guidelines. Coprimary objectives were to assess safety, as occurrence of severe adverse events (AEs), serious AEs (SAEs), and important medical events (IMEs) related to the vaccination, and humoral immunogenicity, as the type 2 seroprotection rate 28 days after the first mOPV2 dose. Secondary objectives included assessment of all SAEs or IMEs and any solicited or unsolicited AEs throughout the study, and humoral immunogenicity to all 3 poliovirus types at 7 and 28 days after each vaccination. An exploratory objective was assessment of viral shedding, later augmented with an evaluation of intestinal immunity at baseline and after each dose.

### Participants

Eligible participants were 1–5-year-old children with a documented history of at least 3 IPV vaccinations in their first year of life, healthy with no obvious medical conditions on examination at enrollment, and with no poliovirus vaccination within the previous 3 months or any other vaccination within the previous 4 weeks. Other exclusion criteria included any known allergy to a vaccine component, any known immunodeficiency or chronic illness, and the presence of anyone in the participant’s household who had received OPV in the previous 3 months.

### Vaccine

The mOPV2 challenge vaccine, Polio Sabin Mono Two (oral) (GlaxoSmithKline Biologicals; lot DOP2A004AZ) is a World Health Organization (WHO)–prequalified, monovalent, live attenuated polio virus vaccine of the Sabin strain type 2 (P712, Ch, 2ab), propagated in MRC5 human diploid cells. Each 0.1-mL dose, delivered as 2 drops from a supplied polyethylene dropper, contained ≥10^5^ 50% cell culture infective dose (CCID_50_) of type 2 poliovirus, magnesium chloride as stabilizer, and trace amounts of neomycin sulfate and polymyxin B sulfate.

### Procedures

Enrolled children were randomized (1:1) to receive 1 dose of mOPV2 or 2 doses, 4 weeks apart. Parents or guardians used mobile phones and remote data entry to record solicited systemic AEs for 7 days after each vaccination, and any unsolicited AEs, including SAEs and IMEs occurring throughout the study duration up to 6 weeks after the final dose of mOPV2. Parents/guardians also supplied weekly stool samples for assessment of viral shedding by real-time reverse-transcription polymerase chain reaction (RT-PCR) [10], augmented with an assessment of intestinal immunity at baseline and after each dose as described elsewhere [11].

### Viral Shedding and Humoral and Intestinal Immunity

Type 2 neutralizing antibodies (NAbs) were measured in serum samples collected before the first dose and 28 days after each vaccination using the WHO standard microneutralization assay (WHO EPI GEN 93.9) at the laboratories of Centers for Disease Control and Prevention in Atlanta, Georgia, as described elsewhere [12]. The application of the assay used there has a lower limit of quantitation (LLOQ) of 2.5 log_2_ titer and an upper limit of quantitation (ULOQ) of 10.5 log_2_ titer. Antibody titers are reported as the reciprocal of the dilution, equating to a range from 5.7 (LLOQ) to 1448 (ULOQ). Geometric mean titers (GMTs) were calculated using the antilog of the arithmetic mean of log_2_ antibody titers with LLOQ and ULOQ as observed values, wherever these values were observed.

After extraction of nucleic acid from stool samples collected at days 0–10, 7, 14, 21, and 28 in groups 1 and 2, and at days 35, 42, and 56 in group 2, poliovirus was detected using RT-PCR [13]. If detected, the titer was measured as CCID_50_.

Intestinal type 2 poliovirus–specific neutralizing activity was measured at Dartmouth University in the same stool samples by limiting dilution inhibition of a luciferase-expressing type 2–specific wild-type–derived polio pseudovirus in vitro, expressed as the titer needed to achieve 60% neutralization, as described elsewhere [14]. Titers <4 were considered undetectable and recorded as 2. Total and poliovirus type 2–specific concentrations of immunoglobulin A (IgA) in stool specimens were assessed relative to a serum standard, using a multiplex Luminex-based microsphere assay developed by coupling monovalent IPVs to fluorescently-coded magnetic microspheres [15], with relative concentration units below the limit of detection (≤10) reported as 5.

### Statistical Analysis

There were 2 coprimary end points—safety and day 28 seroprotection rate—on which the sample size was based. For safety evaluation, a sample size of 45 evaluable participants provided 90% probability of observing ≥1 unexpected AE with true rate of 5%, which was boosted to 50 to allow for 10% dropout before final safety evaluation. For the immunogenicity end point, we assumed a seroprotection rate after a single dose of type 2 OPV in this population of 97%. Recognizing that these data may provide a historical control comparator for development of novel type 2 vaccines, 48 evaluable participants per group would provide 80% power for a hypothetical noninferiority comparison of a new vaccine with these data with type I error rate α = 0.025, and noninferiority margin 10%. Because the safety end point required 50 participants per group, and all 1- and 2-dose participants contribute to the immunogenicity end point, 100 participants were to be enrolled.

Immunogenicity was expressed as seroprotection rate and NAb GMT before and 28 days after each vaccination, and as type 2 seroconversion rates 28 days after each vaccination. Seroprotection rates were defined as group percentages with an NAb titer ≥8. Seroconversion was defined as a change from seronegative to seropositive with a NAb titer ≥8, or in baseline seropositive children, a ≥4-fold increase in antibody titer over baseline. A stool viral shedding index end point (SIE) was calculated as the average of log_10_ CCID_50_ per gram determined using real time RT-PCR (viral identity) and CCID_50_ (titer) from samples obtained on days 7, 14, 21, and 28 after each vaccine dose, and this was summarized descriptively. The per-protocol population, defined as those without major protocol deviations potentially interfering with immunogenicity assessments, was the primary population for analysis.

Pairwise correlations between mOPV2 viral shedding, type 2–specific stool NAb titers, and type 2–specific IgA levels 2 weeks after the first mOPV2 dose were estimated using Spearman rank correlation coefficients. For evaluation of intestinal antibody responses, responders were defined as those achieving a poliovirus type 2–specific stool NAb titer ≥32, and high shedders as those who shed mOPV2 virus with a titer ≥8.25 log10 CCID50 per gram, the ULOQ. The odds of being a high shedder (ie, shedding vaccine virus at the ULOQ) after the second challenge dose, given the shedding status after the first challenge dose, were estimated using logistic regression.

Poliovirus type 2–specific stool neutralization and IgA levels are summarized on day 0 and after mOPV2 challenge as an aggregate of values obtained ±2 days of each target day, presented as a single value representing the mean of the log_10_ CCID_50_ levels or the geometric means of the type 2–specific stool neutralization or IgA if ≥2 stool samples were collected within each 5-day window. Viral shedding titers and poliovirus type 2–specific stool neutralization titers and IgA concentrations were plotted by day after mOPV2 challenge. Intestinal responses were summarized for days 0, 7, 14, 21, and 28 days after the first and second mOPV2 challenges by whether individuals were (1) responders or nonresponders and (2) high shedders or not after the first challenge dose. Distributions across categories were compared using the Mann-Whitney *U* test. All *P* values are from 2-sided statistical tests, and all analyses were performed using Stata software, version 13.0 (StataCorp) and R software, version 3.2.5.

## RESULTS

### Demographics

**Figure 1.**
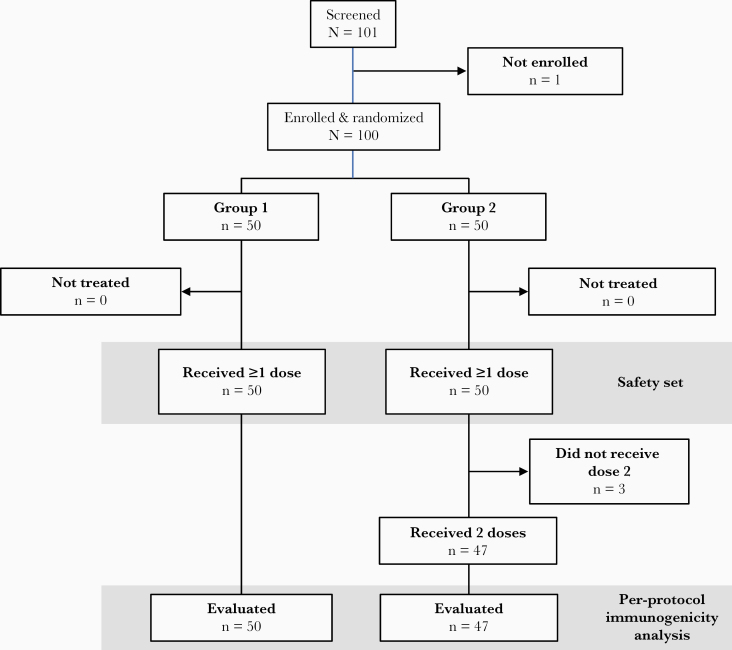
Flow chart of the study.

Of 101 participants screened, 100 were enrolled into the 2 groups (Figure 1). The mean age was 3.0 years (Table 1), and female-male distribution was 45:55. Most children (82%) had received ≥4 prior IPV doses administered in the nationally recommended diphtheria and tetanus toxoids and acellular pertussis–IPV–hepatitis B virus/*Haemophilus influenzae* type b vaccine combination, with 79% having received their most recent vaccination ≥1 year previously. Three children in group 2 did not receive their second mOPV2 so were excluded from the per-protocol population, but all other participants completed all visits according to protocol.

**Table 1.** Demographic Characteristics of the Enrolled Study Population

Characteristic	Group 1 (n = 50)	Group 2 (n = 50)	Total
Sex, No. (%)			
Male	24	31	55
Female	26	19	45
Age, mean (SD), y	3.2 (1.3)	2.8 (1.3)	3.0 (1.3)
Height, mean (SD), cm	100.3 (11.0)	98.1 (11.6)	99.2 (11.3)
Weight, mean (SD), kg	15.8 (3.1)	15.6 (3.1)	15.7 (3.0)
No. of prior IPVs, No (%)			
3	8 (16)	10 (20)	18 (18)
4+	42 (84)	40 (80)	82 (82)
Time since last poliovirus vaccination, No. (%)			
≥1 y	43 (86)	36 (72)	79 (79)
<1 y	7 (14)	14 (28)	21 (21)
Time since last poliovirus vaccination for those vaccinated <1 y earlier, mean (SD), mo	6.4 (2.5)	7.2 (2.7)	6.9 (2.6)

Abbreviations: IPV, inactivated poliovirus vaccine; SD, standard deviation.

### Safety and Tolerability

Safety assessments in 100 children for the first dose and in 47 for the second showed that the mOPV2 challenge was well tolerated (Table 2). One SAE, bronchitis, was observed 21 days after the first dose, resolved after 11 days, and was considered inconsistent with a causal association to immunization. No SAEs, IMEs, or severe AEs were observed with any participant. There were few solicited AEs, which were all mild and inconsistent with causal association to immunization. All unsolicited AEs were reported as mild or moderate and inconsistent with causal association to vaccination, and none led to the withdrawal of a child from the study.

**Table 2.** Safety Assessment: Solicited and Unsolicited Adverse Events^a^

	Reports, No. (%)	
Adverse Events	After Dose 1 (Both Groups; N = 100)	After Dose 2 (Group 2; n = 47)
Any solicited	2 (0)	2 (4)
Fever	1 (1)	0
Abnormal crying	1 (1)	0
Loss of appetite	1 (1)	0
Irritability	1 (1)	1 (2)
Vomiting	0	1 (2)
Any unsolicited	14 (14)	4 (9)
Mild	10 (10)	3 (6)
Moderate	4 (4)	3 (6)
Severe	0	0
Leading to withdrawal	0	0

^**a**^Solicited adverse events in the 7 days after vaccination and unsolicited adverse events throughout the study period. All solicited events were considered mild, and all events were considered inconsistent with a causal association to immunization.

### Humoral Immunogenicity

Humoral immunogenicity assessments were made in 96 children before and after the dose 1, and in 47 after dose 2. These showed a high level of humoral immunity to all 3 polioviruses at baseline, with seroprotection rates of 97%, 98%, and 96% to types 1, 2 and 3, respectively (Table 3). Against this high background, little change could be observed in the seroprotection rates after 1 or 2 doses of mOPV2, although there were some cases of seroconversion in those whose baseline titers were sufficiently below the ULOQ to be evaluable. Seroconversion against type 2 was observed in 29 of 41 evaluable participants (71%) 28 days after 1 dose. No further increase was observed after 2 doses when 11 of 17 evaluable participants (65%) still displayed seroconversion (Table 3). Surprisingly, 9 of 29 (31%) and 3 of 31 (10%) displayed seroconversion to types 1 and 3, respectively, 7 days after 1 dose of mOPV2, suggesting some heterotypic immune response occurred.

**Table 3.** Seroprotection and Seroconversion Rates for the 3 Poliovirus Types after 1 Dose in Combined Groups 1 and 2 and After 2 Doses in Group 2 (Per-Protocol Population)

		Seroprotection or Seroconversion Rate, No./Total (%; 95% CI)					
		Poliovirus Type 1		Poliovirus Type 2		Poliovirus Type 3	
Rate	Day	Groups 1 and 2	Group 2	Groups 1 and 2	Group 2	Groups 1 and 2	Group 2
Seroprotection	0	93/96 (97; 91.1–99.4)	47/47 (100; 92.5–100)	94/96 (98; 92.7–99.7)	47/47 (100; 92.5–100)	92/96 (96; 89.7–98.9)	45/47 (96; 85.5–99.5)
	7	48/50 (96; 86.3–99.5)	…	49/50 (98; 89.4–99.9)	…	49/50 (98; 89.4–99.9)	…
	28	88/90 (98; 92.2–99.7)	45/46 (98; 88.5–99.9)	90/90 (100; 96.0–100)	46/46 (100; 92.3–100)	87/90 (97; 90.6–99.3)	44/46 (96; 85.2–99.5)
	56	…	43/43 (100; 91.8–100)	…	43/42 (100; 91.8–100)	…	41/43 (95; 84.2–99.4)
Seroconversion^a^	7	4/20 (20; 5.7–43.7)	…	9/24 (38; 18.8–59.4)	…	2/19 (11; 1.3–33.1)	…
	28	9/29 (31; 15.3–50.8)	…	29/41 (71; 54.5–83.9)	…	3/31 (10; 2.0–25.8)	…
	56	…	3/10 (30; 6.7–65.2)	…	11/17 (65; 38.3–85.8)	…	1/12 (8; 0.2–38.5)

**Abbreviation: CI, confidence interval.**

^a^Seroconversion **was** calculated **only** for those whose baseline titer was sufficiently low to allow observation of 4-fold increase without exceeding **the u**pper **l**imit of **q**uantitation (ie, baseline titer ≤8.5 log_2_**.)**

**Figure 2.**
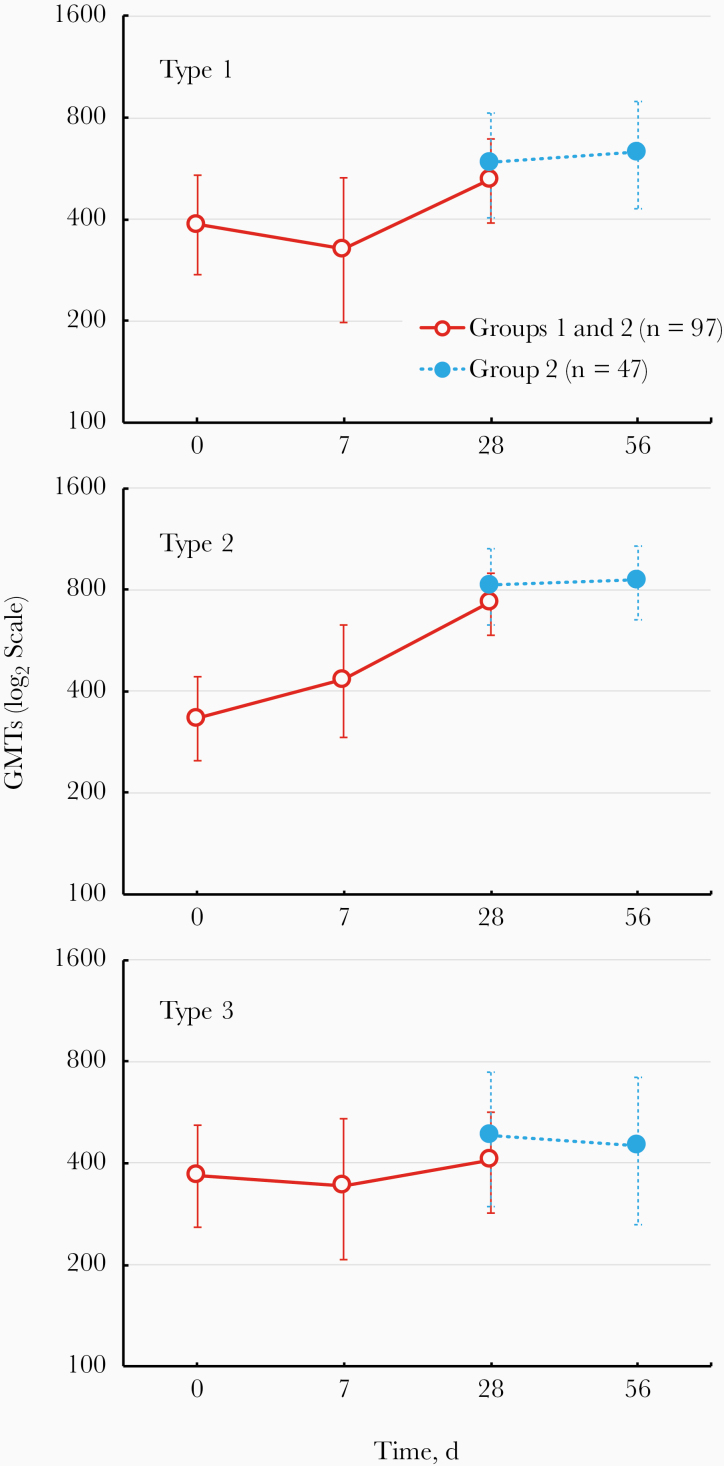
Geometric mean titers (GMTs) (with 95% confidence intervals) of neutralizing antibodies against the 3 poliovirus serotypes in the 2 study groups after 1 (*red symbols*) or 2 (*blue symbols*) challenge doses of and safety of monovalent type 2 oral poliovirus vaccine (mOPV2).

These observations were reflected in the serum NAb GMTs (Figure 2), which were relatively constant for NAbs to types 1 and 3 from day 0 to days 28 and 56, after 1 and 2 mOPV2 doses, also reflecting the frequency of subjects with humoral immunity near the assay ULOQ. Within the limitations of the high baseline, there was evidence of an increase in the type 2 NAb GMT at day 28, with no further increase observed after a second dose of mOPV2. Median log_2_ serum type 2 Nab titers rose from 9.0 (95% confidence interval [CI], 8.2–9.8) to 10.2 (10.2 to ≥10.5) after 1 dose, and from 9.2 (7.5–9.8) to ≥10.5 (9.8 to ≥10.5) among children receiving 2 doses.

### Viral Shedding

**Figure 3.**
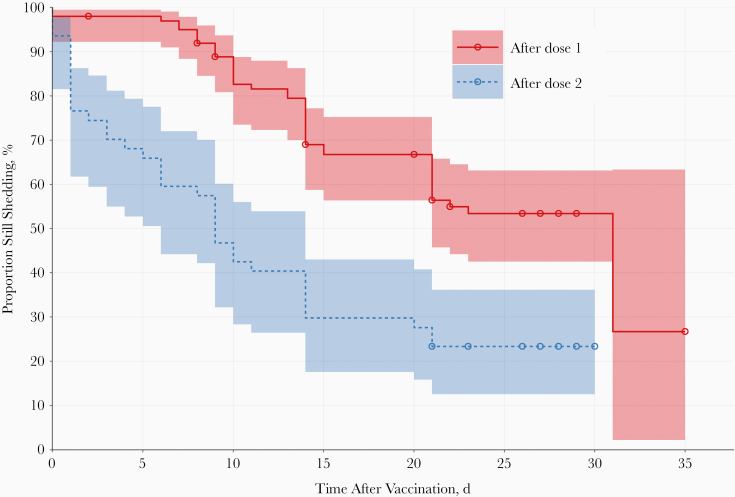
Proportions shedding virus in stool samples over the period after vaccination with monovalent type 2 oral poliovirus vaccine, shown as rate after each dose (with pointwise 95% confidence intervals as shaded areas). The day of cessation was defined as the last day when virus was detected in stool samples, with all following days being negative.

After a single dose, 92% of the 92 evaluable participants had detectable virus (PCR positive) in their stool samples 7 days later, a proportion that declined to 89%, 67%, and 50% on days 14, 21, and 28, respectively (Figure 3). On day 28, there was a median value of 4.83 log_10_ CCID_50_/g (95% CI, 3.69 to ≥8.25) for virus in stool samples from shedders, with 1.38 log_10_ CCID_50_/g (.00–3.19) across all subjects submitting samples at this time point (Table 4). One week after the second dose, 45% of the 44 evaluable participants were shedding vaccine virus, a proportion that declined to 24% (95% CI, 12%–41%) 4 weeks after the second dose with a median value of 3.16 log_10_ CCID_50_/g (95% CI, 2.78 to ≥8.25) among shedders.

**Table 4.** Type 2 Poliovirus Shedding in Stool Samples From Participants After 1 or 2 Doses of Monovalent Type 2 Oral Poliovirus Vaccine (Per-Protocol Population)

Shedding^a^	Groups 1 and 2 After Dose 1 (n = 97)	Group 2 After Dose 2 (n = 47)
Days 7, 14, 21, and 28		
Shedders, No./total (%)	30/31 (97)	11/20 (55)
SIE, median (95% CI), log10 CCID50		
Overall	4.03 (3.11–4.80)	0.69 (.0–2.06)
Among shedders	4.32 (3.24–4.84)	2.06 (.70–3.01)
Day 7		
Shedders, No./total (%)	85/92 (92)	20/44 (45)
Median (95% CI), log10 CCID50		
Overall	5.88 (5.44–6.36)	0 (.0 to ≤2.75)
Among shedders	5.91 (5.72–6.63)	4.42 (2.91–6.09)
Day 14		
Shedders, No./total (%)	55/62 (89)	13/29 (45)
Median (95% CI), log10 CCID50		
Overall	5.11 (4.19–5.72)	0 (.0–3.0)
Among shedders	5.41 (4.59–6.06)	4.69 (3.00 to ≥8.25)
Day 21		
Shedders, No./total (%)	44/66 (67)	10/35 (29)
Median (95% CI), log10 CCID50		
Overall	2.89 (≤2.75 to 3.63)	0 (.0–.0)
Among shedders	4.05 (3.17–5.31)	3.02 (≤2.75 to 4.05)
Day 28		
Shedders, No./total (%)	34/68 (50)	9/37 (24)
Median (95% CI), log10 CCID50		
Overall	1.38 (.0–3.19)	0 (.0–.0)
Among shedders	4.83 (3.69 to ≥8.25)	3.16 (2.78 to ≥8.25)

**Abbreviations: CI, confidence interval; SIE, shedding index end point.**

^a^Rate and extent of viral shedding in polymerase chain reaction–positive participants. The allowable window for stool samples to contribute to the SIE computation was ±2 days from the nominal sampling day.

The median viral SIE values in all analyzed participants were 4.03 after dose 1 and 0.69 after dose 2 (Table 4), illustrating the induction of intestinal immunity by the first dose of mOPV2. When calculated among those with any postdose shedding, the SIEs were 4.32 and 2.06 after doses 1 and 2, respectively, reinforcing the observation of induced intestinal activity.

All 93 children whose stool samples were evaluated for mucosal antibody responses shed vaccine virus during follow-up. Notably, of the 92 children who shed after the first challenge dose, 67 (73%; 95% CI, 63%–82%) were observed to achieve a titer ≥8.25 log_10_ CCID_50_ (ie, the ULOQ) in ≥1 sample. Similarly, of the 32 children who shed after the second challenge dose 20 (63%; 95% CI, 44%–79%) reached a titer ≥8.25 log_10_ CCID_50_ in ≥1 sample. Individuals who shed vaccine virus at the maximum titer after the first dose were >5 times more likely to shed at the maximum titer after the second dose (odds ratio 5.7; 95% CI, 1.3–24.1).

### Intestinal Antibody Responses

Polio type 2–specific IgA concentrations and intestinal neutralizing activity were evaluated in 352 stool samples from 93 children (median age, 3.4 years; interquartile range, 2.8–4.5), in 47 children after 1 mOPV2 challenge dose and in 46 after 2 doses (Supplementary Tables 1 and 2).

IgA was detected in all stool samples tested. The median total IgA concentration in the first stool sample evaluated per child was 6.39 μg/mL (interquartile range, 1.87–12.86 μg/mL). Overall, there was a high degree of concordance between presence of poliovirus type 2 specific stool IgA, neutralizing activity, and diminished shedding. Two weeks after the first mOPV2 dose, there was a strong correlation between poliovirus type 2–specific stool IgA and neutralizing titer (groups 1 and 2, Spearman ρ = 0.90; *P* < .001), and both were negatively correlated with mOPV2 shedding (groups 1 and 2, Spearman ρ = −0.48 for IgA and shedding and −0.47 for neutralizing titer and shedding; both *P* = .003) (Supplementary Figure 1).

Overall, the intestinal antibody responses to mOPV2 challenge were lower than expected (Supplementary Figure 2). Of children with post–dose 1 mucosal antibody responses 28 of 87 (32%; 95% CI, 23%–43%) achieved type 2–specific stool neutralizing titers ≥32 after the first mOPV2 dose. Similarly, 16 of 45 (36%; 95% CI, 22%–51%) with post–dose 2 antibody responses evaluated had titers ≥32; 5 children who did not respond to the first dose had titers ≥32 after the second, and 2 children with titers ≥32 after the first dose had titers <32 after the second. In total, 57 of 93 children (61%; 95% CI, 51%–71%) evaluated did not achieve an intestinal neutralizing activity titer ≥32 at any point during follow-up.

## DISCUSSION

After global cessation of OPV2 use with the switch from trivalent to bivalent OPV in May 2016, the only source of immunity against type 2 polioviruses is from IPV with WHO recommendations to transition to IPV-only schedules when all OPV use has ceased to maintain eradication of all types of polioviruses [16]. Therefore, understanding the impact of IPV-only infant primary schedules on humoral and intestinal protection against polio is critical to inform policy decisions on options for future outbreak response and preparations for eventual cessation of all OPV use. The situation in Lithuania immediately before the switch, with a 3-dose infant primary series of diphtheria and tetanus toxoids and acellular pertussis–hepatitis B virus–IPV/*H. influenzae* type b vaccine and no OPV use in routine immunization or supplementary immunization activities, provided us with a unique opportunity to evaluate intestinal immunogenicity against type 2 in children vaccinated with IPV only after challenge doses of mOPV2. This setting closely reflects the postswitch and postcertification situation and response dynamics to exposure to live type 2 vaccine virus.

A widely accepted surrogate for assessing intestinal mucosal immunity is protection against viral excretion after an oral challenge with the live attenuated vaccine [17]. A recent review of primary intestinal immunogenicity from schedules using IPV as the only source of type 2 poliovirus vaccine found that the majority of vaccinees across most studies shed type 2 virus in stool (60.3%–80.5%) after mOPV2 challenge, similar to prior experience in IPV-only vaccinated subjects receiving an OPV challenge [18]. Interestingly, this review found a marginally favorable impact of 2 IPV doses on both the proportion of participants shedding virus and the overall magnitude of shedding 21 and 28 days after challenge, compared with 1-dose IPV recipients, suggesting some effect of IPV on intestinal immunity. The clinical and epidemiologic relevance of this impact is not known. More recently, the impact of IPV and bivalent OPV on primary intestinal immunity has been assessed by measuring poliovirus-specific antibodies in stool samples obtained from subjects enrolled in randomized controlled trials [11, 19]. These novel assays confirm IPV, both conventional and high dose, has limited impact on intestinal mucosal immune responses or on limiting viral shedding on challenge.

Several studies over the past decades have confirmed the safety and immunogenicity of mOPV2, currently the vaccine of choice for outbreak response for type 2 cVDPVs [20]. The administration of 1 or 2 doses of mOPV2 was well tolerated and safe in this cohort of 1–5-year-olds, with no SAEs or severe AEs associated with the vaccine, an important consideration in the use of mOPV2 in outbreak situations. We confirmed that after 3–4 doses of IPV in infancy, children had high levels of humoral immunity as NAbs against all 3 polioviruses, in addition to which there was still a small but measurable increase in serum NAbs against type 2 poliovirus to achieve a 100% seroprotection rate induced by the first challenge dose of mOPV2. Seroprotection rates were uniformly high at all study time points, precluding observation of changes in this rate, and providing limited ability to evaluate seroconversion. A second dose did not elicit any further increase in serological response. In studies where mOPV2 immunogenicity has been evaluated in naive infants, a clear dose response has been observed with high rates of second-dose seroconversion [21, 22].

When stool samples were assessed by PCR most children had detectable type 2 virus 7 days after exposure to a single vaccine dose of mOPV2, the proportion declining slowly through 28 days after exposure, when half the children were still shedding. Furthermore, 68% of subjects contributing a postvaccination sample were shedding ≥8.25 log_10_ CCID_50_/g of virus within the first 2 weeks after vaccination. Proportions shedding and amounts of shed virus were lower after a second dose of mOPV2, with 24% of children still shedding 28 days later. This indicates that although children had very limited, if any, intestinal immunity from an IPV-only infant immunization schedule, the first mOPV2 vaccination induced some measure of intestinal immunogenicity such that shedding decreased after a second dose of mOPV2. However, the extent of mucosal immunity measured by antibody responses induced by the second dose of mOPV2, at 32%, was relatively poor compared with findings in previous studies where mOPV2 was used as a challenge vaccine in IPV-immunized infants [8].

In the current study, as in others, there was a strong correlation between intestinal poliovirus-specific IgA and neutralization. A study in young Swedish adults who had received IPV in infancy and were challenged with oral monovalent polio type 1 found that they had an even more limited intestinal immune response [23]. The immunologic mechanisms underlying this immune blockade are under active investigation. The current study from Lithuania contributes to our understanding of the limited protection IPV provides against virus shedding after exposure to live virus, reiterating that IPV may not limit child-to-child transmission of circulating wild or VDPVs.

## Supplementary Data

Supplementary materials are available at The *Journal of Infectious Diseases* online. Consisting of data provided by the authors to benefit the reader, the posted materials are not copyedited and are the sole responsibility of the authors, so questions or comments should be addressed to the corresponding author.

jiaa390_suppl_Supplementary_Figure_S1

jiaa390_suppl_Supplementary_Figure_S2

jiaa390_suppl_Supplementary_Legends

jiaa390_suppl_Supplementary_Material

## Notes

***Acknowledgments.*** The authors thank all the parents and guardians of the participants in the study, the study staff at the clinics in Lithuania, and Cassidy L. Papia and Joshua A. Weiner, who performed the mucosal immunoglobulin A analysis. We acknowledge the assistance of Keith Veitch (keithveitch communications) for preparing drafts of the manuscript and providing editorial assistance during the submission.

***Financial support.*** This study was sponsored by the Fighting Infectious Diseases in Emerging Countries (FIDEC) corporation. Grant funding was provided by the Bill & Melinda Gates Foundation.

***Potential conflicts of interest.*** A. S. B. is a salaried employee, and C. G. and R. C. are paid consultants of the Bill & Melinda Gates Foundation, which financed the study. R. R. is a salaried employee of FIDEC, the study sponsor. All other authors report no potential conflicts, other than grant payments to their respective institutions for performing the work. All authors have submitted the ICMJE Form for Disclosure of Potential Conflicts of Interest. Conflicts that the editors consider relevant to the content of the manuscript have been disclosed.

This information has not been presented previously.
