# Supplementary figures and images for: A Randomized Phase 4 Study of Immunogenicity and Safety After Monovalent Oral Type 2 Sabin Poliovirus Vaccine Challenge in Children Vaccinated with Inactivated Poliovirus Vaccine in Lithuania
Source: J Infect Dis. 2020 Jul 4;223(1):119–27. doi: 10.1093/infdis/jiaa390 (PMC7781454; doi:10.1093/infdis/jiaa390)

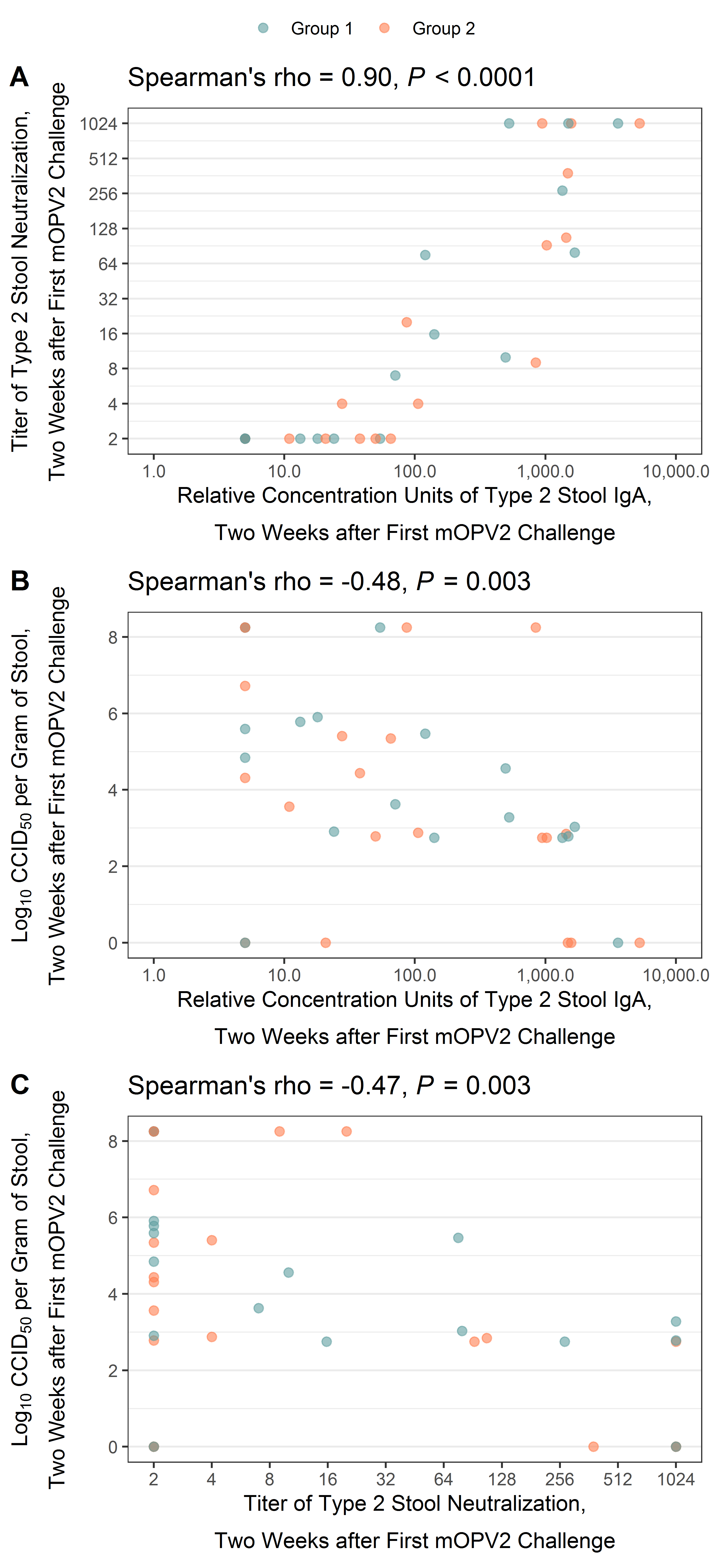

Supplement: jiaa390_suppl_Supplementary_Figure_S1 [file jiaa390_suppl_supplementary_figure_s1.png]

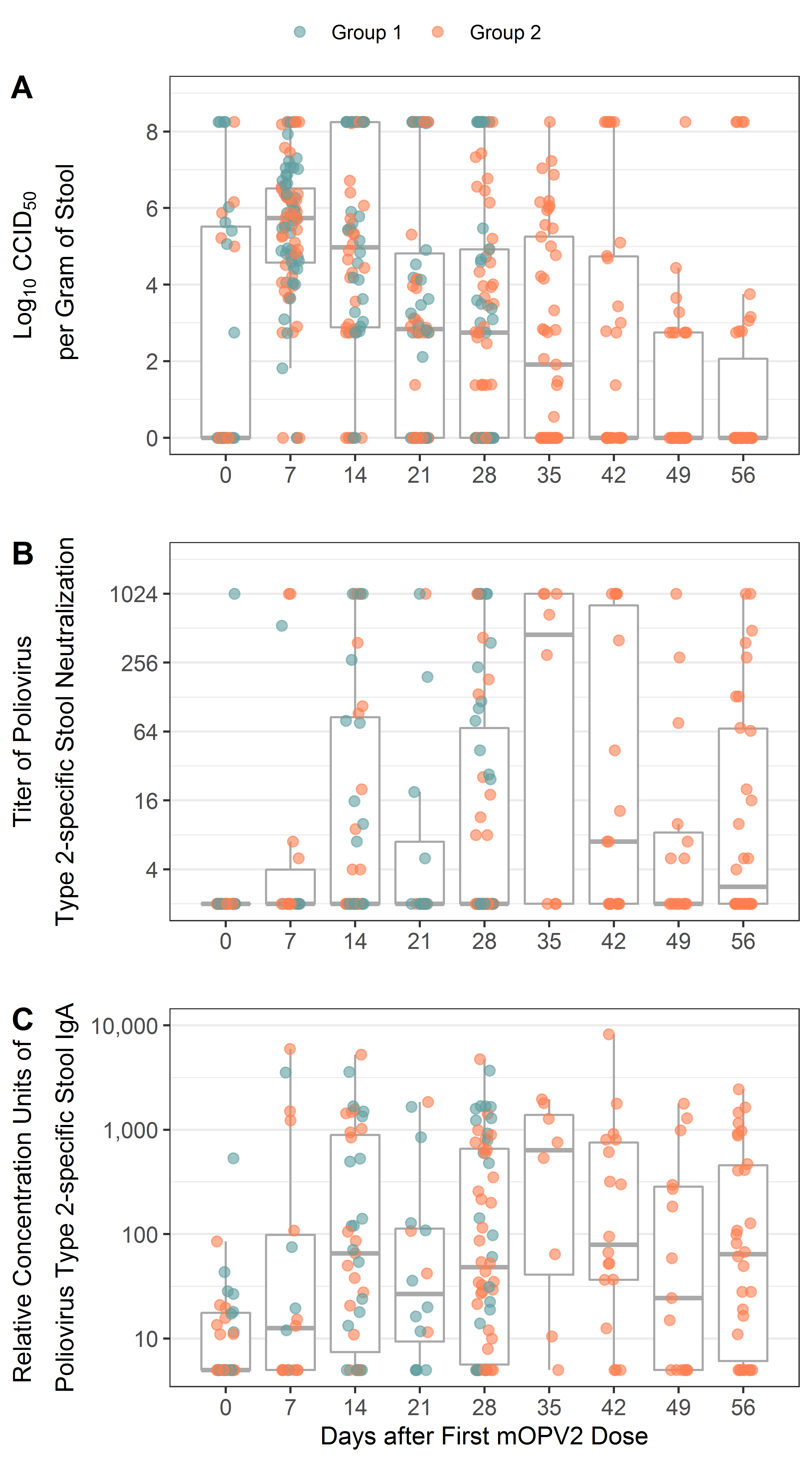

Supplement: jiaa390_suppl_Supplementary_Figure_S2 [file jiaa390_suppl_supplementary_figure_s2.png]
