# Supplementary material for: A Randomized Phase 4 Study of Immunogenicity and Safety After Monovalent Oral Type 2 Sabin Poliovirus Vaccine Challenge in Children Vaccinated with Inactivated Poliovirus Vaccine in Lithuania
Source: J Infect Dis. 2020 Jul 4;223(1):119–27. doi: 10.1093/infdis/jiaa390 (PMC7781454; doi:10.1093/infdis/jiaa390)
Supplement: jiaa390_suppl_Supplementary_Legends [file jiaa390_suppl_supplementary_legends.docx]

**Supplementary Figure 1.**

**Pairwise correlations between poliovirus type 2-specific IgA, neutralization, and shedding two weeks after the first challenge dose of mOPV2.** Abbreviations: mOPV2 = monovalent oral polio vaccine type 2; IgA = immunoglobulin A.

**Supplementary Figure 2.**

**Poliovirus type 2-specific intestinal responses to mOPV2 challenge: (A) mOPV2 shedding, (B) stool neutralization, and (C) stool IgA.** As data were not available for all children across all time points, the boxplot summary values should not be directly compared between time points.

Abbreviations: mOPV2 = monovalent oral polio vaccine type 2; IgA = immunoglobulin A.
