# Supplementary material for: A Randomized Phase 4 Study of Immunogenicity and Safety After Monovalent Oral Type 2 Sabin Poliovirus Vaccine Challenge in Children Vaccinated with Inactivated Poliovirus Vaccine in Lithuania
Source: J Infect Dis. 2020 Jul 4;223(1):119–27. doi: 10.1093/infdis/jiaa390 (PMC7781454; doi:10.1093/infdis/jiaa390)
Supplement: jiaa390_suppl_Supplementary_Material [file jiaa390_suppl_supplementary_material.docx]

**Supplementary Figures and Tables**

**Supplementary Table 1** – Poliovirus type 2 intestinal antibody responses and shedding after the first mOPV2 dose.

**Supplementary Table 2** – Poliovirus type 2 intestinal antibody responses and shedding after the second mOPV2 dose.

**Supplementary Figure 1** – Correlations between poliovirus type 2 intestinal IgA, neutralization activity and shedding after the second mOPV2 dose.

**Supplementary Figure 2** – Poliovirus type 2-specific intestinal responses to mOPV2 vaccination

**Supplementary Table 1.** **Poliovirus type 2-specific intestinal antibody responses and shedding following the first mOPV2 challenge**, stratified by dose 1 responses (i.e., dose 1 responders: poliovirus type 2 stool neutralization titer ≥ 32; dose 1 non-responders: poliovirus type 2 stool neutralization titer < 32) and shedding magnitude (dose 1 high shedders: log10 CCID_50_ ≥ 8.25; dose 1 low shedders: log10 CCID_50_ < 8.25).

|  | **Days** | **Groups 1 & 2, Post-dose 1** | | | | | | | |
| --- | --- | --- | --- | --- | --- | --- | --- | --- | --- |
|  |  | **Dose 1 Stool Neutralizing Antibody Responses** | | | | **Dose 1 mOPV2 Shedding Magnitude** | | | |
|  |  | N | **Dose 1**  **Responders** | **Dose 1**  **Non-responders** | *P value,*  *Mann-Whitney*  *U Test* | N | **Dose 1 High Shedders** | **Dose 1 Low Shedders** | *P value,*  *Mann-Whitney U Test* |
|  |  |  | Median (IQR) | |  |  | Median (IQR) | |  |
| **Type 2 Stool NAbs** | 0 | 20 | 2  (2, 2) | 2  (2, 2) | *0.69* | 24 | 2  (2, 2) | 2  (2, 2) | *0.29* |
|  | 14 | 39 | 271  (76, 1024) | 2  (2, 2) | *<0.0001* | 39 | 2  (2, 20) | 7  (2, 382) | *0.38* |
|  | 28 | 58 | 185  (34.3, 1024) | 2  (2, 2) | *<0.0001* | 62 | 2  (2, 44) | 2  (2, 98.8) | *0.85* |
| **Type 2 Stool IgA** | 0 | 20 | 14.8  (5, 20) | 5  (5, 5) | *0.01* | 24 | 5  (5, 5) | 5  (5, 5) | *0.76* |
|  | 14 | 39 | 1350  (495, 1570) | 19.3  (5, 59.8) | *<0.0001* | 39 | 46.1  (5, 495) | 121  (20.8, 1480) | *0.24* |
|  | 28 | 58 | 806  (288, 1500) | 20  (5, 142) | *<0.0001* | 62 | 48.5  (7.9, 641) | 60.6  (5, 738) | *0.99* |
| **mOPV2 Shedding** | 0 | 22 | 2.2  (0, 5.3) | 0  (0, 7.2) | *0.84* | 26 | 0  (0, 6.2) | 4.3  (0, 5.9) | *0.73* |
|  | 14 | 60 | 3.3  (2.8, 5.6) | 5.3  (3.6, 8.3) | *0.04* | 62 | 5.6  (4.3, 8.25) | 2.8  (0, 3.3) | *<0.0001* |
|  | 28 | 80 | 0  (0, 3.5) | 2.8  (0, 5.8) | *0.07* | 87 | 3.1  (0, 6.5) | 0  (0, 3.5) | *0.08* |

**Supplementary Table 2.**  **Poliovirus type 2-specific intestinal antibody responses and shedding following the second mOPV2 challenge**, stratified by dose 1 responses (i.e., dose 1 responders: poliovirus type 2 stool neutralization titer ≥ 32; dose 1 non-responders: poliovirus type 2 stool neutralization titer < 32) and shedding magnitude (dose 1 high shedders: log10 CCID_50_ ≥ 8.25; dose 1 low shedders: log10 CCID_50_ < 8.25).

|  | **Days** | **Group 2, Post-dose 2** | | | | | | | |
| --- | --- | --- | --- | --- | --- | --- | --- | --- | --- |
|  |  | **Dose 1 Stool Neutralizing Antibody Responses** | | | | **Dose 1 mOPV2 Shedding Magnitude** | | | |
|  |  | N | **Dose 1**  **Responders** | **Dose 1**  **Non-responders** | *P value,*  *Mann-Whitney*  *U Test* | N | **Dose 1 High Shedders** | **Dose 1 Low Shedders** | *P value,*  *Mann-Whitney U Test* |
|  |  |  | Median (IQR) | |  |  | Median (IQR) | |  |
| **Type 2 Stool NAbs** | 0 | 12 | 222  (2, 442) | 2  (2, 2) | *0.40* | 13 | 2  (2, 2) | 2  (2, 18) | *0.28* |
|  | 14 | 16 | 1024  (401, 1024) | 2  (2, 7) | *0.009* | 17 | 2  (2, 401) | 7  (2, 1024) | *0.57* |
|  | 28 | 28 | 256  (34.5, 1024) | 2  (2, 13) | *0.005* | 30 | 4  (2, 20) | 10  (2, 382) | *0.43* |
| **Type 2 Stool IgA** | 0 | 12 | 1515  (34.5, 3200) | 28.0  (5, 200) | *0.19* | 13 | 29.5  (5, 117.5) | 34.5  (29.5, 896) | *0.41* |
|  | 14 | 16 | 806  (612, 1780) | 52.3  (36.5, 94.8) | *0.02* | 17 | 36.7  (12.5, 612) | 183  (52, 805) | *0.43* |
|  | 28 | 28 | 988  (221, 1310) | 55.5  (5, 118) | *0.03* | 30 | 81.5  (28, 408) | 61.4  (5, 1160) | *0.88* |
| **mOPV2 Shedding** | 0 | 14 | 1.6  (0, 3.2) | 3.4  (0, 5.6) | *0.46* | 16 | 2.1  (0.7, 3.0) | 3.4  (0, 5.6) | *0.49* |
|  | 14 | 27 | 1.4  (0, 3.2) | 0  (0, 4.8) | *0.95* | 29 | 3.1  (0, 6.7) | 0  (0, 2.8) | *0.05* |
|  | 28 | 34 | 1.4  (0, 3.2) | 0  (0, 0) | *0.04* | 37 | 0  (0, 0) | 0  (0, 1.4) | *0.97* |
